# Supplementary material for: Iterative improvement in the automatic modular design of robot swarms
Source: PeerJ Comput Sci. 2020 Dec 7;6:e322. doi: 10.7717/peerj-cs.322 (PMC7924708; doi:10.7717/peerj-cs.322)
Supplement: Supplemental Information 3 [file peerj-cs-06-322-s003.zip › argos3/doc/api/standalone/a00527_source.html]

ARGoS: plugins/robots/generic/simulator/gripper\_default\_actuator.h Source File


- Main Page
- Related Pages
- Namespaces
- Classes
- Files

- File List
- File Members

# plugins/robots/generic/simulator/gripper\_default\_actuator.h

Go to the documentation of this file.

```
00001 
00007 #ifndef GRIPPER_DEFAULT_ACTUATOR_H
00008 #define GRIPPER_DEFAULT_ACTUATOR_H
00009 
00010 #include <string>
00011 #include <map>
00012 
00013 namespace argos {
00014    class CGripperDefaultActuator;
00015 }
00016 
00017 #include <argos3/plugins/robots/generic/control_interface/ci_gripper_actuator.h>
00018 #include <argos3/plugins/simulator/entities/gripper_equipped_entity.h>
00019 #include <argos3/core/simulator/actuator.h>
00020 
00021 namespace argos {
00022 
00023    class CGripperDefaultActuator : public CSimulatedActuator,
00024                                    public CCI_GripperActuator {
00025 
00026    public:
00027 
00028       CGripperDefaultActuator();
00029 
00030       virtual ~CGripperDefaultActuator() {}
00031 
00032       virtual void SetRobot(CComposableEntity& c_entity);
00033 
00034       virtual void Update();
00035       virtual void Reset();
00036 
00037    private:
00038 
00039       CGripperEquippedEntity* m_pcGripperEquippedEntity;
00040 
00041    };
00042 
00043 }
00044 
00045 #endif
```

---

Generated on 10 Jul 2018 for ARGoS by 
 1.6.1
